# The seasonal sensitivity of brown bear denning phenology in response to climatic variability

**DOI:** 10.1186/s12983-018-0286-5

**Published:** 2018-11-01

**Authors:** M M Delgado, G Tikhonov, E Meyke, M Babushkin, T Bespalova, S Bondarchuk, A Esengeldenova, I Fedchenko, Y Kalinkin, A Knorre, G Kosenkov, V Kozsheechkin, A Kuznetsov, E Larin, D Mirsaitov, I Prokosheva, Y Rozhkov, A Rykov, I V Seryodkin, S Shubin, R Sibgatullin, N Sikkila, E Sitnikova, L Sultangareeva, A Vasin, L Yarushina, J Kurhinen, V Penteriani

**Affiliations:** 1 Research Unit of Biodiversity (UMIB, UO-CSIC-PA), Oviedo University, Campus Mieres, 33600 Mieres, Spain; 2 University of Helsinki, PO BOX 65, FI-00014 Helsinki, Finland; 3 EarthCape OY, Viikinkaari 6, 00790 Helsinki, Finland; 4 Darwin Nature Biosphere Reserve, Borok, 44 p/o Ploskovo, Cherepovets District, Vologda Region Russian Federation 162723; 5 Kondinskie Lakes National Park, Komsomolski st., 5, City Sovietsky, Hanty-Mansijsk District Russian Federation 628240; 6 Sikhote-Alin State Nature Biosphere Reserve named after K.G. Abramov, Partizanskaya 44, Primorsky krai, Terney Russian Federation 692150; 7 Pinezhsky State Nature Reserve, Pervomayskaya Street, 123 А, Arhangel Region, Pinezkiy District, Pinega Russian Federation 164610; 8 Altai State Nature Biosphere Reserve, Naberezhnyi st., 1, Gorno-Altaysk, Altai Republic Russian Federation 649000; 9 State Nature Reserve Stolby, Kariernaya 26, Krasnoyarsk, Krasnoyarsk Region Russian Federation 660006; 10 Smolenskoe Poozerje National Park, Gurevitch street 19, Demidovskiy District, Przhevalskoe, Smolensk Region Russian Federation 216270; 11 Visimsky Nature Biosphere Reserve, Stepana Razina, 23, Kirovgrad, Russian Federation 624140; 12 State Nature Reserve Malaya Sosva, Lenina str., 46, Sovetskiy, Tjumen Region Russian Federation 628242; 13 State Nature Reserve Vishersky, Gagarina Street 36 B, Krasnovishersk, Perm Region Russian Federation 618590; 14 State Nature Reserve Olekminsky, Filatova 6, Olekminsk, Republic Sakha Russian Federation 678100; 15 Pacific Geographical Institute, Far-Eastern Branch, Russian Academy of Sciences, 7 Radio Street, Vladivostok, Russian Federation 690041; 16 Far Eastern Federal University, 8 Sukhanova Street, Vladivostok, Russian Federation 690091; 17 State Nature Reserve Nurgush, Lenina Street, 129a, Kirov, Russian Federation 610002; 18 Kostomuksha Nature Reserve, Priozernaya Street, 2, Kostomuksha, Karelia Republic Russian Federation 186930; 19 Bryansk Forest Nature Reserve, Nerussa St., Zapovednaya Street, 2, Suzemka District, Bryansk Region Russian Federation 242180; 20 National Park Bashkirija, Nurgush, Abubakirova 1, Meleuzovskiy District, Bashkortostan Republic Russian Federation 453870; 21 Forest Research Institute, Karelian Research Centre, Russian Academy of Sciences, Puskinskaya Street, Petrozavodsk, Russian Federation 11; 22 Pyrenean Institute of Ecology (IPE), C.S.I.C., Avda. Montañana 1005, 50059 Zaragoza, Spain

**Keywords:** Climate change, Denning ecology, Hierarchical Gaussian process, Hibernation, Time-varying coefficients, *Ursus arctos*

## Abstract

**Background:**

For brown bears (*Ursus arctos*), hibernation is a critical part of the annual life cycle because energy savings during hibernation can be crucial for overwintering, and females give birth to cubs at that time. For hibernation to be a useful strategy, timing is critical. However, environmental conditions vary greatly, which might have a negative effect on the functionality of the evolved biological time-keeping. Here, we used a long-term dataset (69 years) on brown bear denning phenology recorded in 12 Russian protected areas and quantified the phenological responses to variation in temperature and snow depth. Previous studies analyzing the relationship between climate and denning behavior did not consider that the brown bear response to variation in climatic factors might vary through a period preceding den entry and exit. We hypothesized that there is a seasonal sensitivity pattern of bear denning phenology in response to variation in climatic conditions, such that the effect of climatic variability will be pronounced only when it occurs close to den exit and entry dates.

**Results:**

We found that brown bears are most sensitive to climatic variations around the observed first den exit and last entry dates, such that an increase/decrease in temperature in the periods closer to the first den exit and last entry dates have a greater influence on the denning dates than in other periods.

**Conclusions:**

Our study shows that climatic factors are modulating brown bear hibernation phenology and provide a further structuring of this modulation. The sensitivity of brown bears to changes in climatic factors during hibernation might affect their ability to cope with global climate change. Therefore, understanding these processes will be essential for informed management of biodiversity in a changing world.

**Electronic supplementary material:**

The online version of this article (10.1186/s12983-018-0286-5) contains supplementary material, which is available to authorized users.

## Background

Animals generally combine internal time-keeping with information from external cues to prepare for predictable, annual changes in their environment (i.e., it is assumed that animals can use environmental information to adjust the timing of various life history activities) [1–3]. Hibernation is an important life history activity that coincides with unfavorable periods (e.g., winter in areas of high latitude) and represents an adaptation for coping with harsh environmental conditions, such as low temperatures and low food abundance [4]. Hibernation is characterized by temporary, pronounced reductions of several physiological functions in heterothermic mammals and represents the most effective means for endotherms to conserve energy through the winter when food supply is limited [4, 5]. The state of torpor in hibernating species might last for several weeks or months, and many individuals can remain more or less continuously in their hibernacula throughout this time [4, 6].

For hibernation to be a useful strategy, it should be both initiated and terminated within specific time frames with respect to environmental factors. The flexibility of the evolved biological clock is thus important to how well animals can cope with varying climatic conditions [7, 8]. In the last few decades, however, environmental conditions (e.g., weather and food availability) have varied greatly, especially under the ongoing climate change [9, 10], which might have a negative effect on the functionality of the evolved biological time-keeping. Changes in phenology have been among the earliest observed ‘footprints’ of global climatic changes [11–13]. However, as most studies describing temporal shifts in phenological dynamics have usually ignored hibernation as a key phenological event for many species [14], our knowledge of whether and to what extent hibernation phenology is shifting in response to changing climatic conditions is limited [9].

For brown bears (*Ursus arctos*), hibernation is a critical period mainly because (a) energy savings during hibernation can be crucial [15, 16] and (b) females give birth to cubs at that time [17]. Even though bears are among the most studied facultative hibernators, mechanisms that drive their denning behavior remain unclear [18]. Here, we used a dataset on the long-term monitoring of brown bear denning phenology (i.e., last den entry and first den exit dates), recorded in 12 Russian localities (i.e., national parks and nature reserves), which spans up to 69 years of observations. To investigate whether climate variability is influencing brown bear denning phenology, we quantified the phenological responses to variation in two climatic variables, temperature and snow depth, which have been previously shown to influence hibernation behavior in brown bears [19, 20] and, more generally, in sedentary mammals [21].

To our knowledge, previous studies assessing the impact of climatic factors on brown bear den exit and entry dates [19, 20] have described and generated predictions for bear denning behavior with respect to climatic data averaged over certain time frames. In contrast, we aimed to assess during which periods of the annual cycle the bears are more sensitive/responsive to climatic variation and, to do this, we evaluated the correlation of climatic variation within a longer period preceding these events. This question is especially relevant under the ongoing warming scenario, which affects climatic characteristics non-uniformly and leads to increased variability over the years and in space [10].

To answer this pertinent research question, we first followed an approach similar to the analysis of Evans et al. [20] to assess the dynamics of temperature and snow around the observed last den entry and first exit dates. Secondly, we specifically looked at how climatic variations in different time frames of the annual cycle are related to the variation in observed last den entry and first exit dates. With that aim, we modeled the last den entry and first exit events with a hierarchical model, where the effects of temperature and snow were included with daily time-varying coefficients. We initially hypothesized that brown bears should be particularly responsive to climatic variation around the typically observed first den exit and last entry dates: a single-unit change in temperature and/or in snow depth in periods closer to these dates might have a greater influence on the observed denning event date in that year than climatic variation at periods further away in time. We further expected that although this temporal pattern might vary from park to park, reflecting bears’ adaptation to local conditions, a common general pattern should emerge. However, we anticipated that the proposed statistical model would better explain variation in first den exit than in last den entry, as previous studies suggested that the timing of den entry is more influenced by other factors, such as the resource availability and an individual’s stored energy [15].

## Methods

### The data

**Fig. 1:**
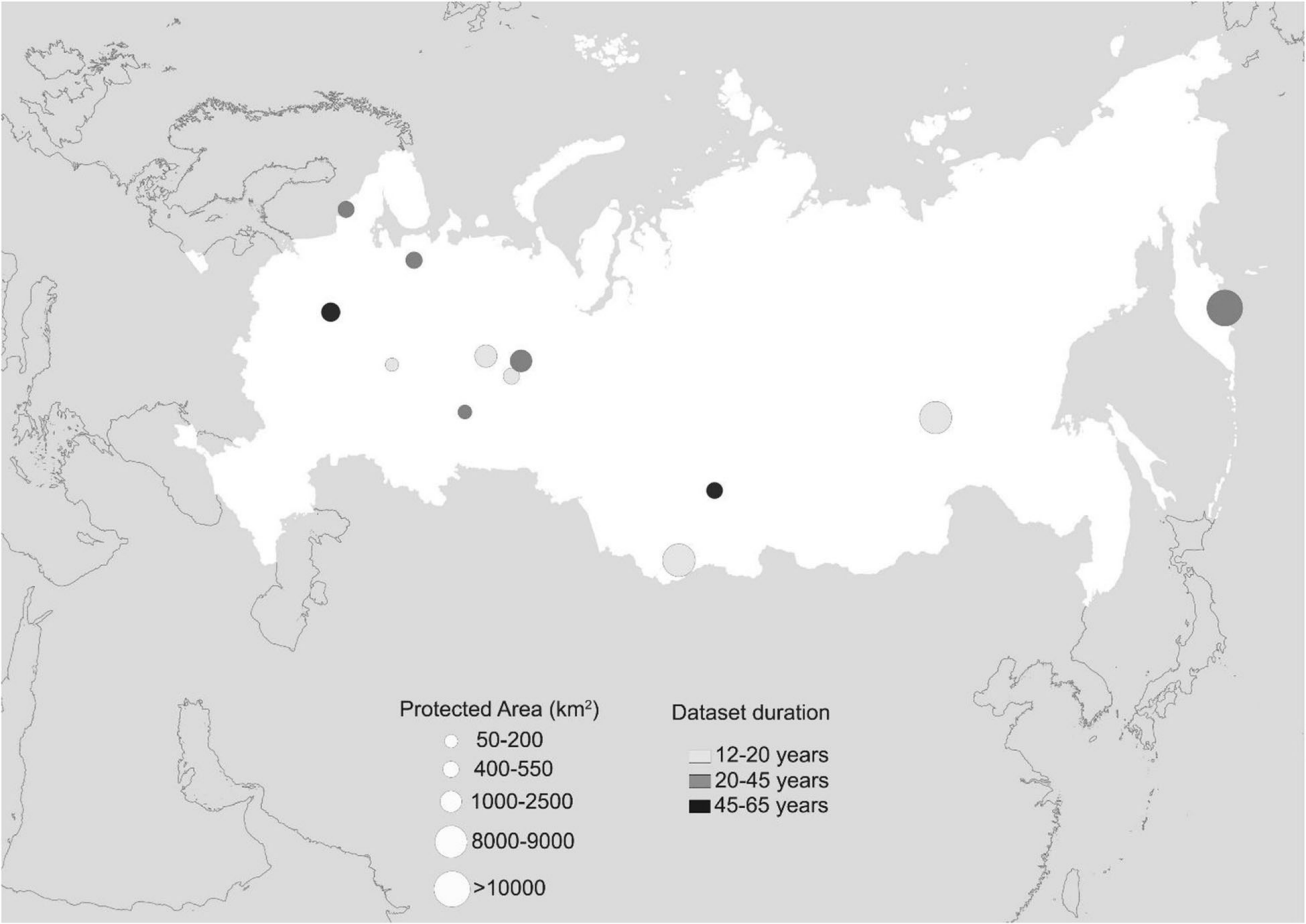
Map showing the distribution of the 12 protected areas distributed throughout Russia. The size of each protected area is represented by different dot sizes, whereas the sampling period of each protected are is represented by different dot colors

The data are part of the “Chronicles of Nature” programme, which was launched in Russia at the end of the 1930s, under which all national parks and nature reserves were required to collect various kinds of biological data in a standardized way [22]. The data on brown bear phenology were collected in 12 natural protected areas (i.e., national parks and nature reserves) located in Russia (Fig. 1); the first time series started in 1946. During this period, researchers conducted systematic fixed route-based observations to record the dates of last/first encounters of bear tracks, which are used as a proxy for dates of last entry/first exit phenological events. Followed routes were designed following landscape structures and always encompassed suitable denning areas, and both direct and indirect (i.e., new footprints and fresh scats) observations were included. Routes were monitored every time a new and fresh snowfall occurred, as well as with constant regularity between snowfalls, and the observed footprints were always marked with a cross. According to weather conditions, routes were monitored all year around either by foot or by snowmobiles. Therefore, we are confident that the last entry and first exit dates were accurately recorded for most of the cases. Notably, as human density in natural protected areas and national parks in Russia is very low, other factors which might potentially affect brown bear hibernation (e.g., human disturbance and food distribution; [23]) were very unlikely to affect the timing of brown bear denning behavior in our study area. Variation in observation effort is of major concern, especially in studies based on volunteer observations. However, even though the number of transects and their length varied across parks, in our dataset the phenological dates were collected in a systematic manner with an approximately constant sampling effort. Given that the variation in sampling effort was present over the study period, we considered this to create additional noise in the data rather than a systematic bias [22].

Also, we used two climatic variables that were recorded on a daily basis in meteorological stations located all around Russia: (1) daily average ambient air temperature and (2) snow depth on the ground. As the locations of the meteorological stations and natural protected areas do not coincide, we interpolated the climatic variables using a nearest-neighbor approach, simply assigning the climatic variables at given protected area with corresponding values from the closest meteorological station (distances from protected areas to nearest station ranged from 13 km to 169 km). We note that for consistency it would be highly beneficial to use meteorological observations exactly from the origin of the phenological data. However, such data has not been recorded on a sufficiently regular basis, and the temporal resolution of available historical reconstructs of climate over the globe is insufficient for our purposes. Thus, we considered these data from meteorological stations to be the best available choice. The meteorological data were provided by All-Russia Research Institute of Hydrometeorological Information - World Data Centre (RIHMI-WDC) and is publicly available on the Institute’s website (http://meteo.ru/data).

### Statistical analyses

We built two separate sets of models to assess the relationship between denning events and climatic factors from two different perspectives. In our first set of models, we tested whether denning events are linked to climatic conditions crossing a particular climatic threshold [20]. We centered a time lag of [− 30, + 30] days around the denning event date observed in a given park and year. We then fitted a hierarchical Gaussian process regression model, where the response variable was the temperature observed in a given park and year each single day within the [− 30, + 30] time lag, and the only explanatory variable was the number of days from each of these days to the center of the time lag (i.e., to the observed denning event date). The formal mathematical specification of the model is.

$$ {v}_{iy}(t)\sim GP\left({u}_i(t),{k}_{3i}\left({t}_1,{t}_2\right)\right)\forall i,y $$

$$ {u}_i(t)\sim GP\left(w(t)+{h}_i,{k}_2\left({t}_1,{t}_2\right)\right)\forall i $$

$$ w(t)\sim GP\left(\mu, {k}_1\left({t}_1,{t}_2\right)\right),t,{t}_1,{t}_2\in \left[-30,+30.\right] $$

In these above equations, *GP*(*f*(*t*), *k*(*t*_1_, *t*_2_)) stands for Gaussian process (GP) with mean function *f*(*t*) and covariance function *k*(*t*_1_, *t*_2_); *v*_*iy*_(*t*) is the observed temperature in park *i* in year *y*, *t* days after the observed denning event (which means |*t*| days before if *t* < 0); *u*_*i*_(*t*) denotes the mean value of temperature in park *i*, *t* days after the observed denning event; *w*(*t*) is the top-level hierarchical mean function of temperature *t* days after the recorded last den entry and first den exit events. Hence, the function *u*_*i*_(*t*) corresponds to the average temperature conditions in park *i* around the denning event date and the function *w*(*t*) corresponds to the global average temperature conditions around the denning event date, also called the population-level estimate. Covariance functions *k*_1_(*t*_1_, *t*_2_) and *k*_2_(*t*_1_, *t*_2_) belong to squared exponential family (reflecting the smoothness of average conditions), and *k*_3*i*_(*t*_1_, *t*_2_) are exponential covariance functions with common range parameter and park-specific variances (reflecting the random-walk pattern of daily temperatures residuals after subtracting the trend and the heteroscedasticity of climate among the parks). Compared to temperature, the snow depths data exhibit much more complicated patterns, involving occasional spiky increases during snowfalls, a slow constant decrease due to compression and vaporization, and a faster decrease due to melting during periods of positive temperatures. The GP-based model described above would utterly fail to replicate these patterns. Therefore, in order to assess the patterns of snow depth variation in the vicinity of denning events, we replaced the statistical modelling with an exploratory analysis that mimics it. In particular, for each park we calculated the average trajectory ${\overline{v}}_i(t)$ of the snow depth in the [−30, +30] time lag by calculating the mean of the snow depth observations ${\widehat{v}}_{iy}(t)$ over the years where denning events were recorded in that park. For each park, we further applied a loess spline smoothing to averaged trajectories ${\overline{v}}_i(t)$ to decrease the amount of empirical noise. We denoted the result of smoothing as ${\widehat{u}}_i(t)$ since they are conceptually equivalent to *u*_*i*_(*t*) in the model built for temperature. Finally, we calculated the global average snow trajectory

$$ \widehat{w}(t)=\frac{1}{N_{parks}}\sum \limits_{i=1}^{N_{parks}}\ {\widehat{u}}_i(t). $$

Second, we built another set of models to analyze the structure of the relationship between observed dates of denning events and climatic conditions, specifically aiming at quantifying the potentially temporally-varying strength of climate effects. We designed an extension of linear mixed models (LMMs), where the response variable was the observed date of the denning event in a given park in a given year, and the explanatory variables included the daily climatic conditions during the season preceding the observed event. Following a memory modeling techniques [24], we assigned Gaussian process priors to the linear regression coefficients to harness the potential of strong temporal autocorrelation in the coefficients. The formal mathematical formulation of this model is

$$ {z}_{iy}=\mu +{r}_i+\sum \limits_{t={t}_0}^T{a}_i(t){u}_{iy}(t)+\sum \limits_{t={t}_0}^T{b}_i(t){v}_{iy}(t)+{\varepsilon}_{yi} $$

$$ {a}_i(t)\sim GP\left(\alpha (t),{k}_a\left({t}_1,{t}_2\right)\right),{b}_i(t)\sim GP\left(\beta (t),{k}_b\left({t}_1,{t}_2\right)\right) $$

$$ \alpha (t)\sim GP\left(0,{k}_{\alpha}\left({t}_1,{t}_2\right)\right),\kern1.25em \beta (t)\sim GP\left(0,{k}_{\beta}\left({t}_1,{t}_2\right)\right) $$

$$ {r}_i\sim Normal\left(0,{\sigma}_r^2\right),\kern1em {\varepsilon}_{yi}\sim Normal\left(0,{\sigma}_i^2\right),\kern1em {\sigma}_i\sim Normal\left(\overline{\sigma},{\rho}^2\right) $$

Here, *z*_*yi*_ is the observed date of a bear denning event in a given park *i* and in year *y*; *u*_*iy*_(*t*) is the function of daily temperature in a given park *i* in a given year *y*; *v*_*iy*_(*t*) is the function of daily snow depths in a given park *i* and in a year *y*. The model parameters include: *μ* – the overall mean day of the selected denning event across all parks; the random intercept component *r*_*i*_ representing variation in mean day in different parks; *α*(*t*)/*β*(*t*) – the time-varying all-parks-common effect of a single unit increase in daily temperature /snow depth on the expected denning event date; *a*_*i*_(*t*)/*b*_*i*_(*t*) – the time-varying *i*-th park-specific effect of a single unit increase in daily temperature /snow on the expected last den entry and first exit dates. The parameter ${\sigma}_i^2$ is the park specific residual variance parameter, also assumed to be hierarchically structured across parks. *k*_*a*_(*t*_1_, *t*_2_) and *k*_*b*_(*t*_1_, *t*_2_) represent two different squared exponential covariance functions, parametrized by common scale, but unique variances; same stands for *k*_*α*_(*t*_1_, *t*_2_) and *k*_*β*_(*t*_1_, *t*_2_). We selected the values for *t*_0_ and *T* such that the interval of [*t*_0_, *T*] covered all recorded dates of the denning event and an additional approximately 50 days before the earliest observed denning event date: *t*_0_ = 211 and *T* = 365 for the last den entry, and *t*_0_ = 1 and *T* = 150 for the first den exit. To assess the quality of our proposed flexible approach, we compared our model with a set of candidate standard LMMs, where the climatic conditions were averaged over all potential time frames [*t*_1_, *t*_2_] (with 7-day step): *t*_1_ = *t*_0_ + 7*k* < *t*_2_ = *t*_0_ + 7*n* ≤ *T*. We calculated the performance of the models via leave-one-out cross-validation (LOO-CV) by taking log-predictive density (LPD) score, which is a natural choice of loss function for probabilistic predictions [25]. Models that yield higher LOO-CV LPD are generally preferred over models showing lower LPD [26].

The two above mentioned sets of models were evaluated following a Bayesian paradigm with numerical computations performed in Stan [27]. Details on assigned priors for hyperparameters, marginal GP representation via covariance function, equivalent computationally-efficient reformulation, Stan code, model fitting, and variance partitioning are provided in the Additional file 1.

## Results

**Fig. 2:**
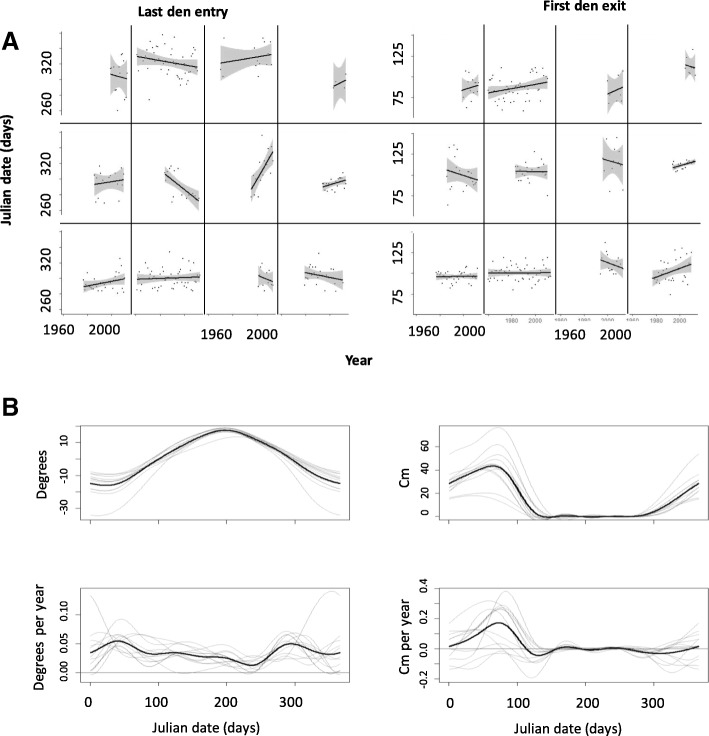
Patterns of phenological and climatic shifts. **a** Phenological shifts for last den entry (left panels) and first den exit (right panels) of the brown bear across Russian national parks and nature reserves. **b** Smoothed seasonal patterns of temperature (°C; left panels) and snow depth (cm; right panels): the upper panels show daily averages over the study period, and the lower panels show the daily mean annual shift (the slope of the linear regression for daily climatic variable vs. year). All patterns were smoothed using cyclic P-splines implemented in the **mgcv** package. Thick black lines stand for the patterns averaged across parks, and each thin grey line depicts a local pattern in a single park. Horizontal red lines correspond to the zero-level of annual shifts

We observed a large variation between locations in the mean timing of bear last den entry and first exit in Russia (Fig. 2a; in Additional file 1: Table S1 and Table S2). In our study area, temperature has experienced a quite uniform increase (on average + 0.034 °C per year; Fig. 2b), whereas snow depth has slightly decreased in autumn but increased in spring (Fig. 2b), possibly as a result of the increase of snowstorms late in the season [10].

**Fig. 3:**
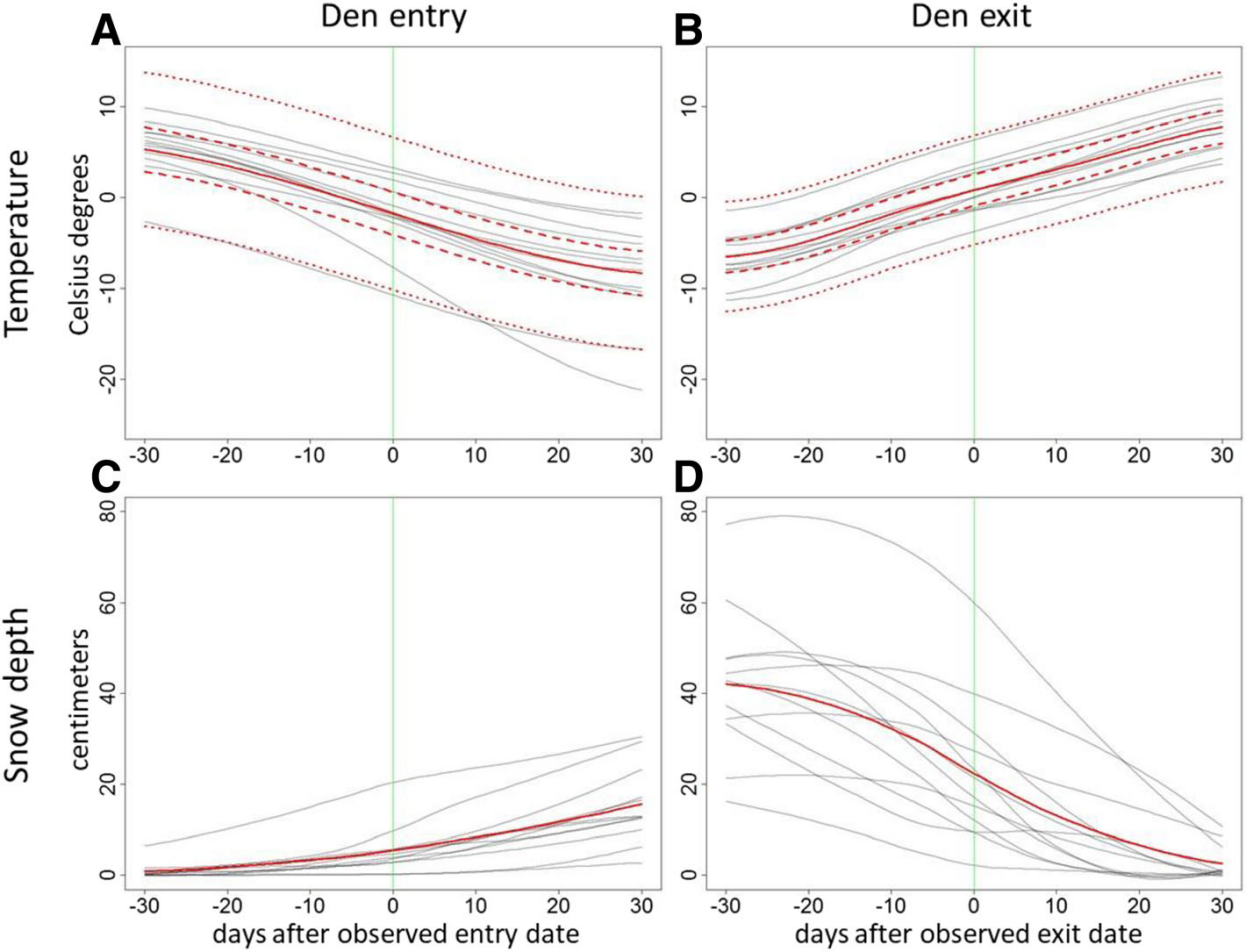
Temperature (**a**, **b**) and snow depth (**c**, **d**) patterns of bear den entry (**a**, **c**) and exit (**b**, **d**) [− 30,+ 30] days around the observed exit/entry dates. The solid red line represents the overall pattern for all studied parks, and gray lines display the park-specific patterns. The results for temperature were derived from a hierarchical Gaussian process regression (for more details, see the first set of models explained in the Statistical analyses) and enabled the uncertainty quantification: dashed red lines represent the 5 and 95% posterior quantiles of the overall pattern *w*(*t*), dotted red lines depict the 5 and 95% posterior quantiles of potential variation in park-specific patterns *u*_*i*_(*t*). The park-specific patterns for snow depth were obtained by fitting loess spline regressions to the long-term empirical averages of daily snow depth observations, and the overall pattern was obtained by taking the mean of site-specific ones

Our first set of models revealed that at the observed last den entry date (mean ± SD = day 304, October 31 ± 18 d; in Additional file 1: Table S1), the expected ambient temperature *w*(0) and snow depth $\widehat{w}(0)$ were − 1.8 °C (±0.03 °C) and 5.5 cm (in Additional file 1: Table S1). We observed that the temperature around this date was, in general, decreasing by 0.3 °C per day (Fig. 3a), and snow depth was simultaneously increasing by 0.25 cm per day (Fig. 3c; in Additional file 1: Table S1). Overall, 28% of the observed variation in temperature was attributed to the generally increasing pattern that was shared by all parks, while park-specific patterns explained an additional 18%; the remaining 54% of the observed variation was attributed to idiosyncratic stochastic variation. Likewise, the partitioning of variation in snow depth around first den exit revealed a shared common pattern (22%), average park-specific trajectories (42%, an additional 20%) and idiosyncratic variation (58%). We further found that at the observed first den exit date (mean ± SD = day 99, April 9 ± 15 d; Additional file 1: Table S2), the expected ambient temperature *w*(0) and snow depth $\widehat{w}(0)$ were 0.8 °C (± 0.04 °C) and 22.4 cm, respectively (Additional file 1: Table S2). The temperature was generally increasing by 0.27 °C per day (Fig. 3b), whereas snow depth was decreasing by 1 cm per day (Fig. 3d; in Additional file 1: Table S2). Overall, of the observed variation in temperature in the considered time lag of [−30, +30] days, 37% were attributed to the generally increasing pattern that was shared by all parks, while park-specific patterns explained an additional 9%; the remaining 54% of the observed variation was attributed to idiosyncratic stochastic variation. Similarly, the partitioning of variation in snow depth around first den exit revealed a shared common pattern (29%), average park-specific trajectories (62%, an additional 33%), and idiosyncratic variation (38%).

**Fig. 4:**
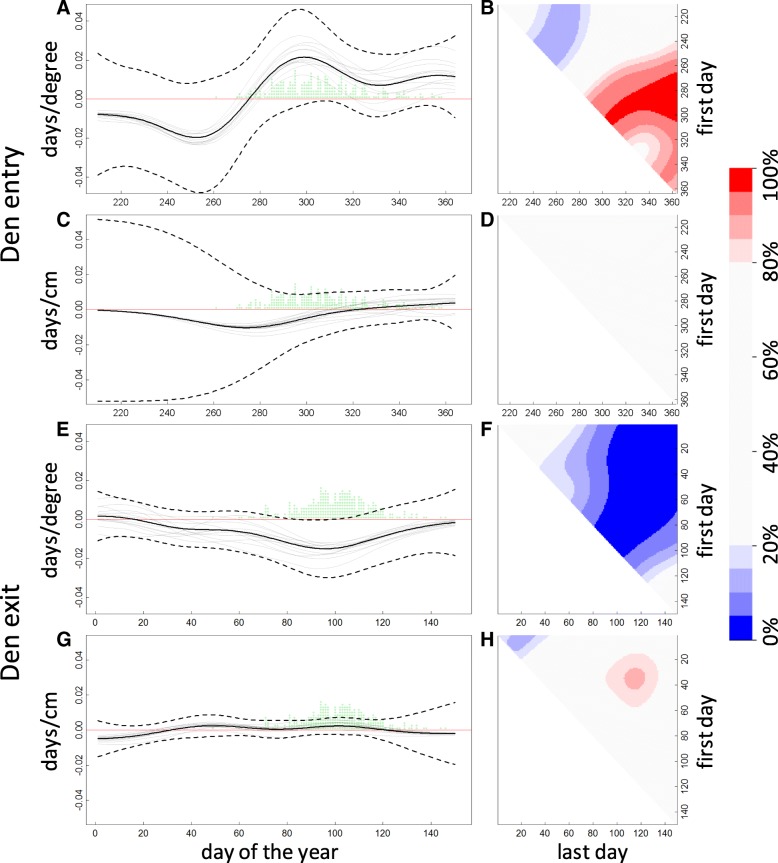
Key model 2 fit results for den entry (**a**, **b**, **c**, **d**) and den exit (**e**, **f**, **g**, **h**). Panels AE depict the posterior mean (solid black line), 5 and 95% quantiles (dashed black lines) for global time-dependent temperature coefficients *α*(*t*), as well as posterior means for park-specific coefficients *a*_*i*_(*t*) (grey lines). Similarly, panels **c**, **g** depict snow depth coefficients *β*(*t*) and *b*_*i*_(*t*). Green dots represent the temporal distribution of dates on which den entry/exit were observed. Panel **b**, **f** depict the posterior credibility for sums $\sum \limits_{t={t}_1}^{t_2}\alpha (t)$ being greater than zero, where *t*_1_ corresponds to the vertical axis, *t*_2_ to horizontal axis, and *t*_0_ ≤ *t*_1_ ≤ *t*_2_ ≤ *T* . Analogously, panels **d**, **h** visualize posterior credibility of $\sum \limits_{t={t}_1}^{t_2}\beta (t)\ge 0$. The credibility colour coding is shown in the colorbar

Our second set of models revealed a pronounced temporally varying structure of the relationship between the last den entry dates and daily temperatures (Fig. 4a, b), whereas the estimate of relation with snow depth remained largely uncertain (Fig. 4c, d). In those years that from late September to early November was warmer than the average, we observed later last den entries. Also, we found that there is an indication that in years with warm late August–early September den exit events occurred earlier than on average. The hierarchical model explained 43.2% of the variation in observed dates; after subtracting the park-specific mean dates, the climate-dependent components explained 10.0%.

Regarding the first den exit, we found a profound relationship between spring temperatures and the first observed exit dates (Fig. 4e, f), whereas the snow depth exhibited a weak relationship (Fig. 4g, h). We observed that in those years where the temperatures in March–mid-April were higher than average, first den exit events were observed earlier (Fig. 4f). Furthermore, for each day from day 81 to day 104, the daily temperature had a negative correlation with first den exit events (*P*_*post*_(*α*(*t*) < 0) > 0.95). That is, when temperature was warmer in any of these days and other climate variables were unchanged, first den exit dates were expected to be observed earlier. The hierarchical model explained 46.6% of variation in observed first dates; after subtracting the park-specific mean dates, the climate-dependent components explained 17.1%.

Our flexible linear mixed model outperformed the traditional linear mixed modeling. LOO-CV of our model resulted in − 1053.8 and − 1097.5 in terms of LPD scores for last den entry and first den exit respectively, whereas the tested candidate LMMs with differently selected time-spans for climatic variables averaging were at best − 1076.3 and − 1134.0 for last den entry and first den exit, respectively.

## Discussion

Seasonal timing of animal hibernation has changed in response to climate change [21, 28, 29]. We additionally confirmed that climatic factors had likely been modulating brown bear hibernation phenology and quantified the patterns of such modulation. In particular, a warmer year earlier in spring than average local conditions triggered earlier brown bears first den exit. As the temperature is expected to increase by at least 1.5 °C between the 20th and 21st centuries [10, 30], previous findings have suggested that bears will indeed emerge from dens earlier as the climate continues to warm [14, 15]. However, the complex interactions between intrinsic and extrinsic factors governing hibernation [31] make it difficult to isolate the impact of climatic factors in observational studies.

Climate-induced changes in the phenology of bear hibernation could result in energy stress, reduced cub survival and fitness [15] and increased human-bear conflicts [32]. As has been observed in other hibernating mammals [33–35], temperatures above the upper threshold of optimal hibernation temperature range could increase bear metabolic rate, thus increasing consumption of stored energy reserves. Changes in denning behavior, notably early emergence, could result in reduced fitness of individuals [36] and/or trophic mismatches might occur when spring food resources are still unavailable. Indeed, since brown bears are sensitive to temperature, expected warmer climate might reduce the duration of hibernation, which might cause trophic mismatches for those individuals that emerge from dens in the presence of abundant snow that drastically reduces the available spring food resources [28]. Early den exit might also have negative consequences on the condition of cubs at den exit [37] and might further increase human-bear conflicts associated with unseasonably warm springs or autumns, as expected with future climate conditions [14, 32]. Thus, human-bear conflicts might increase as a result of changing bear behavior, irrespective of brown bear population sizes [14]. Yet, if higher spring temperatures due to climate change result in food becoming available earlier, a correspondingly earlier den exit might be advantageous for brown bears. Indeed, in many Southern areas, brown bears are well-adapted not to hibernate, with no apparent negative consequences at the individual and the population levels [38, 39]. Therefore, whether and to what extent changes in brown bear denning behavior due to climate change will affect individual fitness and, consequently, brown bear populations, is still an open pertinent question.

Previous studies analyzing the association between climate and denning behavior have not considered that the denning events might be influenced by climate conditions over a long preceding period, with the influence of climatic factors potentially varying temporally. We found that there is a temporally-varying sensitivity pattern of brown bear denning phenology in response to variation in climatic conditions, with the relationship to climatic variation being more pronounced closer to the average date that bears first exit their dens. For example, if the average daily temperature increases by 1 °C compared to average local conditions in the period of 10–30 days before the overall mean day of first den exit (day 99), our model showed that bears emerged from their dens 0.3 days earlier. The same increase occurring 50–70 days before the overall mean first day of den exit would have much less influence, if any at all. This result is important because ongoing global warming is altering the mean temperature and precipitation in a non-uniform way over the year [10, 40]. We could, for example, observe 1 year characterized by a warm mean temperature in which, close to the end of hibernation, the temperature might exhibit a sudden decrease compared to average local conditions. As cold temperatures close to den exit may delay this event, we might erroneously suggest that a warmer spring leads to later dates of den exit if we consider the general mean spring temperature alone. As climatic variability likely impacts energy balance, phenology, and cold damage through effects on metabolism and development [41], species-specific sensitivity to changes in climatic variability might be particularly important in determining organisms’ responses to climate change [40].

Our findings suggest that an important cue triggering the completion of bear hibernation is the variation in spring temperature, especially when it occurs close to average den exit dates. However, bear phenological plasticity in response to climate change is also supposed to be driven by other climatic factors, such as snow depth [21, 42]. The complex feedback mechanisms and interactions between temperature and snow depth make it difficult to predict changes in bear hibernation in response to temporal variation in climate. Changes in the absolute variability of these factors (as well as their synchrony) can modify the interaction and outcomes of snow depth and temperature on bear denning behavior. For example, mean air temperatures are increasing globally, but the historically positive correlation between warmer springs and earlier snow melting dates is now disappearing in many areas due to the increase of spring snowstorms late in the season [10]. Notably, our analyses reveal the importance of considering local conditions before accounting for the influence of snow depth in particular years. Many local factors, from intrinsic local climate stochasticity to changes in local climatic conditions that were not captured in our predictor data, certainly influence bear denning behavior.

In contrast to that previously suggested by Craighead and Craighead [43], and recently observed by Evans et al. [20], we found no relationship between snow depth and first den entry, and only a limited relationship between autumn temperature and first den entry. Time-series data have indicated that the primary causes of first den entry for hibernation are food availability and early snowfall [23], although studies from different regions have come to different conclusions about their effects [19, 44–46]. This suggests that the initiation of hibernation might show high flexibility in response to local conditions. From our data, however, it is not possible to clearly discriminate whether the lack of climate influence is due to the intrinsic stochasticity of this phenological event or the inherent difficulties in measuring den entry dates via the last bear encounter. While we agree that, for example, averaged den entry/exit dates would provide a more robust assessment of phenological change than dates on last/first occurrences, such data are simply not available for the long period and spatially extensive area considered in this study. Nevertheless, the long-term and spatially extensive data we are presenting here are crucial for improving our understanding of phenological responses to climate change. It is important to note, however, that the current state-of-the-art of global positioning system (GPS) telemetry technology would allow us to collect much more precise and comprehensive information on brown bear denning behavior [20]. GPS-based radiotelemetry studies are essential in our search for a mechanistic understanding of key concepts of animal ecology [47], including brown bear phenological responses to climatic change. Extended use of brown bear remote tracking over long periods of time and over large spatial scales can provide robust inferences for complex, multi-factorial phenomena, such as the effects of climate change on brown bear ecology and behavior.

The two modeling approaches we applied go beyond the traditional analytical tools previously applied to analyze phenological responses to climatic variations. While focusing on the same phenomenon, our frameworks are fine-tuned for quantifying different types of relationship between climatic variables and phenological event. The first set of models, based on Evans et al. [20], aimed at capturing the interdependence of observed phenological events and climatic variables passing certain thresholds. The second set of models was focused on estimating the temporarily varying additive effects of temperature and snow on the phenological dates. However, further improvement for the phenological analysis would involve combining these two approaches together into one model. Another very computationally intensive and data-demanding, but highly-valuable, extension would be to adapt survival modeling techniques to honestly model the dependence of bear activity only on past information. Apart of multiple modeling benefits, such as the ability to potentially distinguish the effects of generally confounded variables (e.g., increasing temperature and photoperiod prior to den exit date), this would also allow us to make the important practical advance from assessing the correlative nature of relations to inferring causal dependence.

## Conclusions

Our study shows that the timing of brown bear denning behavior seems to be more influenced by climatic variation happening close to the average entry/exit dates. In the last few decades, environmental conditions have varied greatly under ongoing climate change, which might have a negative effect on the functionality of the evolved biological time-keeping. As hibernation is a critical part of the brown bear annual life cycle, their sensitivity during this period to changes in climatic factors might affect their ability to cope with global climate change. Therefore, understanding these processes will be essential for informed management of biodiversity in a changing world as climate-induced changes in hibernation have the potential to affect individual and population fitness [21].

## Additional file

Additional file 1: **Table S1 and S2A**. Complete formal description of applied statistical analyses, including the codes of the models and a summary of data and descriptive statistics. (DOCX 45 kb)

## Abbreviations

GP: Gaussian Process
LMMs: Linear mixed models
LOO-CV: Leave-one-out cross-validation
LPD: Log-predictive density
