# Supplementary material for: The seasonal sensitivity of brown bear denning phenology in response to climatic variability
Source: Front Zool. 2018 Nov 1;15:41. doi: 10.1186/s12983-018-0286-5 (PMC6211405; doi:10.1186/s12983-018-0286-5)
Supplement: Supplementary file 1 — Table S1 and S2A. Complete formal description of applied statistical analyses, including the codes of the models and a summary of data and descriptive statistics. (DOCX 45 kb) [file 12983_2018_286_MOESM1_ESM.docx]

Statistical analysis

# Model 1

## Full model specification

The full Bayesian specification of model 1 follows:

$$v_{iy}\left( t \right) \sim GP\left( u_{i}\left( t \right), k_{3i}\left( t_{1},t_{2} \right) \right)$$

$$u_{i}\left( t \right) \sim GP\left( w\left( t \right)+h_{i}, k_{2}\left( t_{1},t_{2} \right) \right)$$

$$w\left( t \right) \sim GP\left( \mu, k_{1}\left( t_{1},t_{2} \right) \right)$$

$$k_{1}\left( t_{1},t_{2} \right)=\sigma_{1}^{2}\exp\left( \frac{\left| t_{1}-t_{2} \right|^{2}}{l_{1}^{2}} \right)$$

$$k_{2}\left( t_{1},t_{2} \right)=\sigma_{2}^{2}\exp\left( \frac{\left| t_{1}-t_{2} \right|^{2}}{l_{2}^{2}} \right)$$

$$k_{3i}\left( t_{1},t_{2} \right)=\sigma_{3i}^{2}\exp\left( \frac{\left| t_{1}-t_{2} \right|}{l_{3}} \right)$$

$$\mu\sim N\left( 0,\sigma_{\mu}^{2} \right), h_{i}\sim N\left( 0,\sigma_{h}^{2} \right)$$

$$\sigma_{\mu}^{2}={10}^{4} , \sigma_{h}^{2}=25, \sigma_{1}^{2}\sim N_{+}\left( {0,10}^{2} \right), \sigma_{2}^{2}\sim N_{+}\left( {0,10}^{2} \right), \sigma_{3i}^{2}\sim N_{+}\left( {0,10}^{2} \right)$$

$$l_{1}\sim N_{+}\left( {30,10}^{2} \right), l_{2}\sim N_{+}\left( {30,10}^{2} \right), l_{3}\sim N_{+}\left( {4,2}^{2} \right)$$

where $N_{+}\left( \mu,\sigma^{2} \right)$ stands for the positive-only truncation of normal distribution with mean $\mu$ and variance $\sigma^{2}$.

## Marginal Gaussian process representation

Gaussian process (GP) and Gaussian terms in the above formulation of the model 1 could be marginalized to obtain a marginal representation of the model as a Gaussian process with following covariance function:

$$cov\left( v_{iy}\left( t \right),v_{i^{'}y^{'}}\left( t' \right) \right)=\sigma_{\mu}^{2}+k_{1}\left( t,t' \right)+\delta_{ii^{'}}\left( \sigma_{h}^{2}+k_{2}\left( t,t' \right) \right)+\delta_{ii^{'}}\delta_{yy^{'}}k_{3i}\left( t,t' \right)$$

where $\delta_{ij}$ is the Kronecker delta. This marginal representation is highly beneficial for the model fitting, since only hyperparameters of the model must be estimated numerically, which drastically reduces the complexity of posterior sampling and makes it much easier to devise/tune an efficient sampling algorithm. However, the direct GP approach to computations is critically hampered by the high-dimensionality of observations: 61 daily observations per park-year pair and 288 pairs for den exit (259 for den entry) result in $N=$ 17500 observations. As the numerical complexity of direct GP methods scales as $N^{3}$ (calculation of for Gaussian likelihood involves computation of quadratic form with precision matrix and determinant of covariance matrix), this makes the computations practically infeasible. To overcome this issue, we exploited the composite structure of covariance matrix and used Woodbury matrix identity to derive a better-scaling algorithm based on for computing the desired quadratic form and determinant. Given the applied ecological context of the main text, we leave the technical and rather cumbersome derivations of the resulted formulas and present only the results.

## Stan program

The resulted model can be encoded with Stan probabilistic programming language:

*data***{**

**int<**lower**=**1**>** Y**;**

**int<**lower**=**1**>** D**;**

**int<**lower**=**1**>** P**;**

**int** m**[**P**];**

**matrix[**D**,**Y**]** ZA**[**P**];**

**vector[**D**]** zS**[**P**];**

**real<**lower**=**0**>** sd1**;**

**real<**lower**=**0**>** sd2**;**

**real<**lower**=**0**>** sd3**;**

**real<**lower**=**0**>** sigmaM**;**

**real<**lower**=**0**>** sigmaE**;**

**real<**lower**=**0**>** sdH**;**

**}**

*transformed* *data***{**

**matrix[**D**,**D**]** dist**;**

**real** indDay**[**D**];**

**for(**i **in** 1**:**D**)**

**for(**j **in** 1**:**D**)**

dist**[**i**,**j**]** **=** abs**(**i**-**j**);**

**for(**i **in** 1**:**D**)**

indDay**[**i**]** **=** i**;**

**}**

*parameters***{**

**real<**lower**=**0**>** sigma1**;**

**real<**lower**=**0**>** sigma2**;**

**real<**lower**=**0**>** sigma3**[**P**];**

**real<**lower**=**0**>** l1**;**

**real<**lower**=**0**>** l2**;**

**real<**lower**=**0**>** l3**;**

**real<**lower**=**0**>** sigmaH**;**

**}**

*model***{**

**matrix[**D**,**D**]** K1**;**

**matrix[**D**,**D**]** K2**;**

**matrix[**D**,**D**]** K3**[**P**];**

**matrix[**D**,**D**]** iK1**;**

**matrix[**D**,**D**]** iK2**;**

**matrix[**D**,**D**]** iK3**[**P**];**

**matrix[**D**,**D**]** A**;**

**matrix[**D**,**D**]** B**;**

**matrix[**D**,**D**]** iB**;**

**vector[**D**]** v**;**

**real** qF**;**

**real** logDet**;**

K1 **=** sigmaM **+** sigma1*****exp**(-(**dist**/**l1**)**.***(**dist**/**l1**))** **+** diag_matrix**(**rep_vector**(**sigmaE**,**D**));**

K2 **=** sigmaH **+** sigma2*****exp**(-(**dist**/**l2**)**.***(**dist**/**l2**))** **+** diag_matrix**(**rep_vector**(**sigmaE**,**D**));**

iK1 **=** inverse_spd**(**K1**);**

iK2 **=** inverse_spd**(**K2**);**

**for(**p **in** 1**:**P**){**

K3**[**p**]** **=** sigma3**[**p**]***exp**(-(**dist**/**l3**));**

iK3**[**p**]** **=** inverse_spd**(**K3**[**p**]);**

**}**

iB **=** iK1**;**

logDet **=** 0**;**

qF **=** 0**;**

v **=** rep_vector**(**0**,**D**);**

**for(**p **in** 1**:**P**){**

A **=** inverse_spd**(**quad_form_sym**(**iK2**,**K3**[**p**])+**m**[**p**]***K3**[**p**]);**

iB **+=** m**[**p**]***iK3**[**p**]** **-** m**[**p**]^**2 ***** A**;**

logDet **+=** log_determinant**(**iK2**+**m**[**p**]***iK3**[**p**])** **+** log_determinant**(**K2**)** **+** m**[**p**]***log_determinant**(**K3**[**p**]);**

qF **+=** trace_quad_form**(**iK3**[**p**],** ZA**[**p**])** **-** quad_form_sym**(**A**,**zS**[**p**]);**

v **+=** **(**iK3**[**p**]-**m**[**p**]***A**)** ***** zS**[**p**];**

**}**

B **=** inverse_spd**(**iB**);**

logDet **+=** log_determinant**(**iB**)** **+** log_determinant**(**K1**);**

qF **-=** quad_form_sym**(**B**,**v**);**

**target** **+=** -0.5***(**qF **+** logDet**);**

sigma1 **~** **normal(**0**,**sd1**);**

sigma2 **~** **normal(**0**,**sd2**);**

sigma3 **~** **normal(**0**,**sd3**);**

l1 **~** **normal(**30**,**10**);**

l2 **~** **normal(**30**,**10**);**

l3 **~** **normal(**4**,**2**);**

sigmaH **~** **normal(**0**,**sdH**);**

**}**

This model was fitted using the NUTS sampler with default adaptation parameters and random initiation. We ran 4 chains and 2000 iterations in each chain, out of which first 1000 were used for adaptation and the later 1000 were included to the resulted posterior. The model fit diagnostics indicated proper within and between chain mixing.

## Model fit summary and diagnostics

Model fit summary for temperature around den entry events:

Inference for Stan model: model3.

4 chains, each with iter=2000; warmup=1000; thin=1;

post-warmup draws per chain=1000, total post-warmup draws=4000.

mean se_mean sd 2.5% 25% 50% 75% 97.5% n_eff Rhat

sigma1 16.86 0.09 5.58 7.84 12.86 16.26 20.11 29.64 4000 1

sigma2 10.15 0.08 4.01 4.08 7.27 9.57 12.41 19.84 2705 1

sigma3[1] 31.43 0.03 1.68 28.29 30.28 31.38 32.53 34.83 4000 1

sigma3[2] 34.42 0.02 1.13 32.29 33.63 34.40 35.16 36.64 2672 1

sigma3[3] 74.63 0.06 3.34 68.35 72.29 74.61 76.87 81.15 3398 1

sigma3[4] 14.77 0.02 1.56 11.98 13.68 14.69 15.74 18.21 4000 1

sigma3[5] 14.46 0.01 0.68 13.17 14.00 14.46 14.91 15.83 4000 1

sigma3[6] 41.80 0.03 1.80 38.38 40.55 41.77 42.98 45.46 3079 1

sigma3[7] 35.76 0.03 1.66 32.56 34.65 35.68 36.82 39.23 3083 1

sigma3[8] 55.85 0.05 2.51 51.16 54.10 55.77 57.51 60.94 2990 1

sigma3[9] 23.31 0.02 0.91 21.56 22.70 23.30 23.91 25.17 2852 1

sigma3[10] 46.38 0.03 1.49 43.61 45.35 46.35 47.33 49.49 2309 1

sigma3[11] 36.97 0.03 2.01 33.24 35.60 36.91 38.30 41.04 4000 1

sigma3[12] 32.82 0.02 1.39 30.28 31.87 32.78 33.73 35.79 4000 1

l1 40.28 0.13 7.57 25.36 35.18 40.17 45.47 55.02 3493 1

l2 46.59 0.12 7.12 33.33 41.54 46.41 51.26 61.23 3488 1

l3 5.25 0.00 0.13 5.02 5.17 5.25 5.34 5.51 1632 1

sigmaH 6.85 0.05 3.22 1.15 4.49 6.70 8.96 13.62 4000 1

lp__ -26859.78 0.07 3.05 -26866.63 -26861.63 -26859.45 -26857.62 -26854.82 1730 1

Samples were drawn using NUTS(diag_e) at xxxxx 2018.

For each parameter, n_eff is a crude measure of effective sample size,

and Rhat is the potential scale reduction factor on split chains (at

convergence, Rhat=1).

Model fit summary for temperature around den exit events:

Inference for Stan model: model3.

4 chains, each with iter=2000; warmup=1000; thin=1;

post-warmup draws per chain=1000, total post-warmup draws=4000.

mean se_mean sd 2.5% 25% 50% 75% 97.5% n_eff Rhat

sigma1 17.02 0.09 5.47 8.18 13.00 16.38 20.49 29.15 4000 1

sigma2 4.11 0.04 1.97 1.46 2.68 3.71 5.16 9.09 2588 1

sigma3[1] 29.41 0.03 1.48 26.64 28.35 29.37 30.40 32.50 3206 1

sigma3[2] 28.21 0.02 0.92 26.48 27.58 28.20 28.83 30.04 2553 1

sigma3[3] 40.61 0.04 2.48 36.06 38.88 40.48 42.22 45.69 4000 1

sigma3[4] 18.58 0.02 1.26 16.26 17.72 18.53 19.37 21.17 4000 1

sigma3[5] 12.35 0.01 0.53 11.40 11.98 12.34 12.70 13.41 4000 1

sigma3[6] 41.23 0.03 1.72 38.01 40.09 41.18 42.36 44.72 3052 1

sigma3[7] 25.98 0.02 1.36 23.41 25.03 25.93 26.86 28.81 4000 1

sigma3[8] 26.53 0.02 1.23 24.17 25.68 26.50 27.34 28.99 2848 1

sigma3[9] 27.02 0.02 1.00 25.10 26.32 27.00 27.70 29.06 2810 1

sigma3[10] 38.93 0.02 1.23 36.55 38.09 38.93 39.75 41.41 2579 1

sigma3[11] 31.79 0.03 1.51 28.92 30.77 31.73 32.77 34.98 3276 1

sigma3[12] 29.92 0.02 1.15 27.74 29.13 29.89 30.69 32.28 3124 1

l1 31.66 0.10 5.91 22.08 27.73 30.91 34.69 45.48 3513 1

l2 46.55 0.15 7.83 31.38 41.28 46.49 51.68 62.16 2905 1

l3 5.08 0.00 0.12 4.85 5.00 5.08 5.17 5.33 1647 1

sigmaH 4.56 0.04 2.49 0.57 2.74 4.29 6.02 10.07 4000 1

lp__ -28824.65 0.08 3.08 -28831.49 -28826.50 -28824.35 -28822.38 -28819.61 1671 1

Samples were drawn using NUTS(diag_e) at xxxxx 2018.

For each parameter, n_eff is a crude measure of effective sample size,

and Rhat is the potential scale reduction factor on split chains (at

convergence, Rhat=1).

## Variance partitioning

We partitioned the variance of observed temperature data into three groups that reflect the hierarchical nature of model 1: explained by common pattern, explained by park-specific patterns and unexplained. We denoted the sample variance of observed data as $V_{0}$, residual sample variance after accounting for common hierarchical mean function $w\left( t \right)$ by $V_{w}$ and residual sample variance after accounting for park-specific functions $u_{i}\left( t \right)$ by $V_{u}$. Then we calculated the proportion of variance explained by common pattern as $1-\frac{V_{w}}{V_{0}}$, the proportion of variance explained by park-specific patterns as $1-\frac{V_{u}}{V_{0}}$, and the proportion of unexplained variance as $\frac{V_{u}}{V_{0}}$.

Calculation of variance partitioning for snow depths followed the same formulas as for temperature with the exception that the  $\hat{w}\left( t \right)$ and  $\hat{u}_{i}\left( t \right)$ were calculated not via statistical modelling, but with a descriptive approach that mimics it (presented in the main text).

| Partitioning of climatic variables’ variance in [-30,+30] time-span around the observed denning events | | | | | | |
| --- | --- | --- | --- | --- | --- | --- |
|  | temperature | | | snow depth | | |
|  | common | park-specific | unexplained | common | park-specific | unexplained |
| Den entry | 28% | 46% | 54% | 22% | 42% | 58% |
| Den entry | 37% | 46% | 54% | 29% | 62% | 38% |

# Model 2

## Full model specification

The full Bayesian specification of model 2 follows:

$$z_{iy}=\mu+r_{i}+\sum_{t=t_{0}}^{T} a_{i}\left( t \right)u_{iy}\left( t \right) +\sum_{t=t_{0}}^{T} b_{i}\left( t \right)v_{iy}\left( t \right)+\varepsilon_{yi}$$

$$a_{i}\left( t \right) \sim GP\left( \alpha\left( t \right),k_{a}\left( t_{1},t_{2} \right) \right), b_{i}\left( t \right) \sim GP\left( \beta\left( t \right),k_{b}\left( t_{1},t_{2} \right) \right)$$

$$\alpha\left( t \right) \sim GP\left( 0,k_{\alpha}\left( t_{1},t_{2} \right) \right), \beta\left( t \right) \sim GP\left( 0,k_{\beta}\left( t_{1},t_{2} \right) \right)$$

$$r_{i}\sim N\left( 0, \sigma_{r}^{2} \right), \varepsilon_{yi} \sim N\left( 0, \sigma_{i}^{2} \right), \sigma_{i} \sim N\left( \bar{\sigma}, \rho^{2} \right)$$

$$k_{a}\left( t_{1},t_{2} \right)=\sigma_{a}^{2}\exp\left( \frac{\left| t_{1}-t_{2} \right|^{2}}{l_{2}^{2}} \right), k_{b}\left( t_{1},t_{2} \right)=\sigma_{a}^{2}\exp\left( \frac{\left| t_{1}-t_{2} \right|^{2}}{l_{2}^{2}} \right)$$

$$k_{\alpha}\left( t_{1},t_{2} \right)=\sigma_{\alpha}^{2}\exp\left( \frac{\left| t_{1}-t_{2} \right|^{2}}{l_{1}^{2}} \right), k_{\beta}\left( t_{1},t_{2} \right)=\sigma_{\beta}^{2}\exp\left( \frac{\left| t_{1}-t_{2} \right|^{2}}{l_{1}^{2}} \right)$$

$$\sigma_{a}^{2} \sim N_{+}\left( {0,0.01}^{2} \right), \sigma_{b}^{2} \sim N_{+}\left( {0,0.01}^{2} \right), \sigma_{\alpha}^{2} \sim N_{+}\left( {0,0.01}^{2} \right), \sigma_{\beta}^{2} \sim N_{+}\left( {0,0.01}^{2} \right)$$

$$l_{1} \sim N_{\left[ 3,\infty\right]}\left( {40,10}^{2} \right), l_{2} \sim N_{\left[ 3,\infty\right]}\left( {40,10}^{2} \right), \sigma_{r}\sim N_{+}\left( {0,3}^{2} \right), \bar{\sigma}\sim N_{+}\left( {0,10}^{2} \right), \rho=5$$

Parameter was fixed to the empirical mean of $z_{iy}$: $\mu=305.6$for den entry model and $\mu=100.1$ for den exit model. $N_{+}\left( \mu,\sigma^{2} \right)$ and $N_{\left[ a,b \right]}\left( \mu,\sigma^{2} \right)$ stand for the positive-only truncation and truncation to $\left[ a,b \right]$ interval of normal distribution with mean $\mu$ and variance $\sigma^{2}$.

In terms of likelihood function, the model 2 is invariant to potentially heterogeneous shifts in climatic variables $u_{iy}\left( t \right)$ and $v_{iy}\left( t \right)$ once it is compensated by adjusting the $r_{i}$ terms. However, we would like the $r_{i}$ terms to have tractable meaning, so that we can assume a meaningful hierarchical prior on them. For this purpose, we used not the raw temperature and snow depth observations, but their deviations from long-term averages over the years. In this case, the climatic variables $u_{iy}\left( t \right)$ and $v_{iy}\left( t \right)$ are centred on zero $\forall i,t$over the years and the a priory expectation of $\sum_{t=t_{0}}^{T} a_{i}\left( t \right)u_{iy}\left( t \right) +\sum_{t=t_{0}}^{T} b_{i}\left( t \right)v_{iy}\left( t \right)$ is zero $\forall i$. This leads to a natural interpretation of the $r_{i}$ terms – these are the random intercepts in the model, which tell on how much earlier or later denning events generally happen in given park than on average over the whole study area.

## Marginal Gaussian process representation

Gaussian process (GP) and Gaussian terms in the above formulation of the model 2 could be marginalized to obtain a marginal representation of the model as a Gaussian process with following covariance function:

$$cov\left( z_{iy},z_{i^{'}y^{'}} \right)=\boldsymbol{u}_{iy}^{T}K_{\alpha}\boldsymbol{u}_{i^{'}y^{'}}+\boldsymbol{v}_{iy}^{T}K_{\beta}\boldsymbol{v}_{i^{'}y^{'}}+\delta_{ii^{'}}\left( \sigma_{r}^{2}+\boldsymbol{u}_{iy}^{T}K_{a}\boldsymbol{u}_{i^{'}y^{'}}+\boldsymbol{v}_{iy}^{T}K_{b}\boldsymbol{v}_{i^{'}y^{'}} \right)+\delta_{ii^{'}}\delta_{yy^{'}} \sigma_{i}^{2}$$

where $\delta_{ij}$ is the Kronecker delta,

$$\boldsymbol{u}_{iy}=\left[ u_{iy}\left( t_{0} \right),\ldots,u_{iy}\left( T \right) \right]^{T}, \boldsymbol{v}_{iy}=\left[ v_{iy}\left( t_{0} \right),\ldots,v_{iy}\left( T \right) \right]^{T}$$

$$K_{\alpha}=\left[ k_{\alpha}\left( t_{1},t_{2} \right) \right]_{t_{1}=t_{0}\ldots T}^{t_{2}=t_{0}\ldots T}, K_{\beta}=\left[ k_{\beta}\left( t_{1},t_{2} \right) \right]_{t_{1}=t_{0}\ldots T}^{t_{2}=t_{0}\ldots T}$$

$$K_{a}=\left[ k_{a}\left( t_{1},t_{2} \right) \right]_{t_{1}=t_{0}\ldots T}^{t_{2}=t_{0}\ldots T}, K_{b}=\left[ k_{b}\left( t_{1},t_{2} \right) \right]_{t_{1}=t_{0}\ldots T}^{t_{2}=t_{0}\ldots T}$$

This marginal representation is highly beneficial for the model fitting, since only hyperparameters of the model must be estimated numerically, which drastically reduces the complexity of posterior sampling and makes it much easier to devise/tune an efficient sampling algorithm. Furthermore, it supports highly efficient matrix notation that leads to optimized numerical performance.

## Stan program

The resulted model can be encoded with Stan probabilistic programming language:

*data***{**

**int<**lower**=**1**>** N**;**

**int<**lower**=**1**>** yN**;**

**int<**lower**=**1**>** dN**;**

**int<**lower**=**1**>** pN**;**

**vector[**N**]** day**;**

**int** park**[**N**];**

**int** year**[**N**];**

**matrix[**N**,**dN**]** temp**;**

**matrix[**N**,**dN**]** snow**;**

**real<**lower**=**0**>** sd1**;**

**real<**lower**=**0**>** sd2**;**

**}**

*transformed* *data***{**

**matrix[**dN**,**dN**]** dist2**;**

**int** pLen**[**pN**];**

**int** pInd**[**pN**,**dN**];**

**vector[**N**]** zeros **=** rep_vector**(**0**,**N**);**

**matrix[**dN**,**N**]** tempT **=** temp**';**

**matrix[**dN**,**N**]** snowT **=** snow**';**

**for(**i **in** 1**:**dN**)**

**for(**j **in** 1**:**dN**)**

dist2**[**i**,**j**]** **=** abs**(**i**-**j**)^**2**;**

**for(**p **in** 1**:**pN**)**

pLen**[**p**]** **=** 0**;**

**for(**i **in** 1**:**N**){**

pLen**[**park**[**i**]]** **=** pLen**[**park**[**i**]]** **+** 1**;**

pInd**[**park**[**i**],**pLen**[**park**[**i**]]]** **=** i**;**

**}**

**}**

*parameters***{**

**real<**lower**=**0**>** sigEpsMean**;**

**vector<**lower**=**0**>[**pN**]** sigEps**;**

**real<**lower**=**0**>** sigR**;**

**real<**lower**=**0**>** sigT1**;**

**real<**lower**=**0**>** sigT2**;**

**real<**lower**=**0**>** sigS1**;**

**real<**lower**=**0**>** sigS2**;**

**real<**lower**=**3**>** l1**;**

**real<**lower**=**3**>** l2**;**

**}**

*model***{**

**matrix[**dN**,**dN**]** D1**;**

**matrix[**dN**,**dN**]** D2**;**

**matrix[**dN**,**dN**]** Kt1**;**

**matrix[**dN**,**dN**]** Kt2**;**

**matrix[**dN**,**dN**]** Ks1**;**

**matrix[**dN**,**dN**]** Ks2**;**

**matrix[**N**,**N**]** H**;**

**matrix[**N**,**N**]** H2**;**

D1 **=** exp**(-**dist2 **/** l1**^**2**);**

D2 **=** exp**(-**dist2 **/** l2**^**2**);**

Kt1 **=** sigT1 ***** D1**;**

Kt2 **=** sigT2 ***** D2**;**

Ks1 **=** sigS1 ***** D1**;**

Ks2 **=** sigS2 ***** D2**;**

H2 **=** rep_matrix**(**0**,**N**,**N**);**

**for(**p **in** 1**:**pN**){**

**int** parkInd**[**pLen**[**p**]];**

parkInd **=** pInd**[**p**,**1**:**pLen**[**p**]];**

H2**[**parkInd**,**parkInd**]** **=** sigR**^**2 **+** quad_form**(**Kt2**,**tempT**[,**parkInd**])** **+** quad_form**(**Ks2**,**snowT**[,**parkInd**])** **+** diag_matrix**(**rep_vector**(**sigEps**[**p**]^**2**,**pLen**[**p**]));**

**}**

H **=** quad_form**(**Kt1**,**tempT**)** **+** quad_form**(**Ks1**,**snowT**)** **+** H2**;**

day **~** **multi_normal(**zeros**,** H**);**

sigEpsMean **~** **normal(**0**,**10**);**

sigEps **~** **normal(**sigEpsMean**,**5**);**

sigR **~** **normal(**0**,**3**);**

sigT1 **~** **normal(**0**,**sd1**);**

sigT2 **~** **normal(**0**,**sd1**);**

sigS1 **~** **normal(**0**,**sd2**);**

sigS2 **~** **normal(**0**,**sd2**);**

l1 **~** **normal(**40**,**10**);**

l2 **~** **normal(**40**,**10**);**

**}**

This model was fitted using the NUTS sampler with default adaptation parameters and random initiation. We ran 4 chains and 20000 iterations in each chain, out of which first 10000 were used for adaptation and the later 10000 were additionally thinned by 10 and included to the resulted posterior. The model fit diagnostics indicated proper within and between chain mixing.

## Model fit summary and diagnostics

Model fit summary for den entry events:

Inference for Stan model: modelM1.

4 chains, each with iter=20000; warmup=10000; thin=10;

post-warmup draws per chain=1000, total post-warmup draws=4000.

mean se_mean sd 2.5% 25% 50% 75% 97.5% n_eff Rhat

sigEpsMean 14.10 0.03 1.63 10.89 13.01 14.11 15.20 17.32 4000 1

sigEps[1] 20.03 0.04 2.78 15.21 18.07 19.77 21.79 25.90 4000 1

sigEps[2] 17.57 0.03 1.80 14.50 16.27 17.44 18.65 21.51 4000 1

sigEps[3] 17.73 0.05 2.90 12.76 15.65 17.55 19.45 24.08 4000 1

sigEps[4] 12.13 0.07 4.17 5.29 9.02 11.70 14.82 21.30 3949 1

sigEps[5] 16.14 0.04 2.59 11.67 14.29 15.93 17.71 21.82 3836 1

sigEps[6] 16.27 0.04 2.64 11.77 14.41 16.14 17.90 21.98 4000 1

sigEps[7] 20.58 0.04 2.66 15.99 18.68 20.38 22.28 26.30 3882 1

sigEps[8] 5.66 0.02 1.42 3.48 4.65 5.44 6.45 9.14 4000 1

sigEps[9] 9.74 0.03 1.61 7.10 8.59 9.56 10.73 13.32 3836 1

sigEps[10] 11.59 0.02 1.24 9.41 10.73 11.47 12.34 14.52 4000 1

sigEps[11] 13.15 0.04 2.70 8.73 11.22 12.90 14.75 19.21 4000 1

sigEps[12] 12.35 0.03 2.09 8.96 10.86 12.10 13.63 17.06 3709 1

sigR 8.01 0.02 1.32 5.68 7.11 7.91 8.80 10.81 3867 1

sigT1 0.00 0.00 0.00 0.00 0.00 0.00 0.00 0.01 3785 1

sigT2 0.00 0.00 0.00 0.00 0.00 0.00 0.00 0.00 4000 1

sigS1 0.00 0.00 0.00 0.00 0.00 0.00 0.00 0.01 3814 1

sigS2 0.00 0.00 0.00 0.00 0.00 0.00 0.00 0.00 4000 1

l1 40.00 0.15 9.67 21.64 33.52 39.83 46.38 59.59 4000 1

l2 35.82 0.18 10.66 14.32 28.59 35.84 43.05 56.61 3709 1

lp__ -833.75 0.05 3.30 -840.99 -835.81 -833.43 -831.33 -828.36 3820 1

Samples were drawn using NUTS(diag_e) at xxxxx 2018.

For each parameter, n_eff is a crude measure of effective sample size,

and Rhat is the potential scale reduction factor on split chains (at

convergence, Rhat=1).

Model fit summary for den exit events:

Inference for Stan model: modelM1.

4 chains, each with iter=20000; warmup=10000; thin=10;

post-warmup draws per chain=1000, total post-warmup draws=4000.

mean se_mean sd 2.5% 25% 50% 75% 97.5% n_eff Rhat

sigEpsMean 11.68 0.03 1.59 8.53 10.63 11.70 12.77 14.79 3584 1

sigEps[1] 13.45 0.04 2.45 9.50 11.71 13.16 14.85 19.08 4000 1

sigEps[2] 13.40 0.03 1.53 10.74 12.32 13.30 14.36 16.66 3707 1

sigEps[3] 15.14 0.05 2.95 10.12 13.00 14.90 17.01 21.62 3924 1

sigEps[4] 11.93 0.05 3.13 6.85 9.63 11.62 13.81 19.01 3279 1

sigEps[5] 16.26 0.04 2.43 12.02 14.54 16.12 17.80 21.39 4000 1

sigEps[6] 10.39 0.03 1.73 7.52 9.16 10.20 11.49 14.22 3782 1

sigEps[7] 18.38 0.05 2.85 13.32 16.37 18.24 20.21 24.26 4000 1

sigEps[8] 4.58 0.02 1.04 2.95 3.88 4.42 5.14 7.02 4000 1

sigEps[9] 5.84 0.01 0.82 4.48 5.25 5.75 6.32 7.73 4000 1

sigEps[10] 8.07 0.01 0.87 6.54 7.45 8.01 8.60 9.96 3962 1

sigEps[11] 11.91 0.04 2.26 8.29 10.28 11.66 13.28 17.02 4000 1

sigEps[12] 14.12 0.03 1.95 10.72 12.69 14.00 15.33 18.51 4000 1

sigR 7.54 0.02 1.25 5.36 6.66 7.45 8.32 10.28 4000 1

sigT1 0.00 0.00 0.00 0.00 0.00 0.00 0.00 0.00 3988 1

sigT2 0.00 0.00 0.00 0.00 0.00 0.00 0.00 0.00 3836 1

sigS1 0.00 0.00 0.00 0.00 0.00 0.00 0.00 0.00 4000 1

sigS2 0.00 0.00 0.00 0.00 0.00 0.00 0.00 0.00 3980 1

l1 40.75 0.17 10.68 19.67 33.64 40.75 48.07 61.47 4000 1

l2 33.48 0.18 11.60 6.72 26.94 34.27 41.36 54.61 4000 1

lp__ -859.95 0.05 3.32 -867.31 -861.96 -859.56 -857.60 -854.62 4000 1

Samples were drawn using NUTS(diag_e) at xxxxx 2018.

For each parameter, n_eff is a crude measure of effective sample size,

and Rhat is the potential scale reduction factor on split chains (at

convergence, Rhat=1).

## Competitor LMM models

We assessed the quality of the proposed flexible approach and compared it to results that can be achieved with traditional linear mixed effects models (LMM). As LMM candidates, we tested the following class of LMM that could be fitted with lme4 R package:

$$z_{iy}=r_{i}+a_{i}\frac{\sum_{t=t_{1}}^{t_{2}} u_{iy}\left( t \right)}{t_{2}-t_{1}+1} +b_{i}\frac{\sum_{t=t_{1}}^{t_{2}} v_{iy}\left( t \right)}{t_{2}-t_{1}+1}+\varepsilon_{yi}, \varepsilon_{yi}\sim N\left( 0,\sigma^{2} \right),$$

where we iterated over all potential time frames (with 7-day step) for averaging climatic variables: $t_{1}=t_{0}+7k<t_{2}=t_{0}+7n\leq T$. We calculated models’ performance via leave-one-out cross-validation (LOO-CV) taking log-predictive density, which is a natural choice for probabilistic predictions.

Supplementary Tables

## Table 1. Summary of data and descriptive statistics for last den entry

| Locality | N | Mean date (range) | Mean Temperature (°C) | Shift Temperature (°C/day) | Mean Snow (cm) | Shift Snow (cm/day) |
| --- | --- | --- | --- | --- | --- | --- |
| Altajskij | 13 | 304 (261-334) | 1.92 | -0.23 | 6.4 | 0.11 |
| Darvinskij | 45 | 323(275-358) | -2.20 | -0.21 | 4.2 | 0.23 |
| Kondinskiye Ozera | 14 | 329 (305-354) | -10.75 | -0.29 | 19.7 | 0.61 |
| Kostomukshskij | 3 | 293 (290-298) | 3.28 | -0,24 | 0.3 | 0.03 |
| Kronockij | 16 | 297 (270-317) | 2.70 | -0.20 | 0 | 0 |
| Malaja Sos'va | 18 | 293 (271-317) | -2.77 | -0.33 | 10 | 0.84 |
| Nurgush | 16 | 311 (278-357) | -0.90 | -0.28 | 6.2 | 0.22 |
| Olekminskij | 18 | 295 (284-309) | -7.70 | -0.54 | 4.9 | 0.33 |
| Pinezhskij | 28 | 295 (282-326) | 0.62 | -0.26 | 4.5 | 0.14 |
| Stolby | 49 | 301 (285-335) | -2.19 | -0.35 | 2.8 | 0.17 |
| Visherskij | 11 | 301 (285-320) | -1.62 | -0.34 | 1.5 | 0.13 |
| Visimskij | 22 | 305 (284-333) | -1.93 | -0.31 | 3.4 | 0.21 |

## Table 2. Summary of data and descriptive statistics for first den exit

| Locality | N | Mean date (range) | Mean Temperature (°C) | Shift Temperature (°C/day) | Mean Snow (cm) | Shift Snow (cm/day) |
| --- | --- | --- | --- | --- | --- | --- |
| Altajskij | 14 | 88 (64-108) | 0.75 | 0.20 | 14.9 | -0.84 |
| Darvinskij | 48 | 87 (49-112) | 0.03 | 0.25 | 21.2 | -1.29 |
| Kondinskiye Ozera | 8 | 85 (69-108) | -3.74 | 0.27 | 42.6 | -1.00 |
| Kostomukshskij | 8 | 113 (103-134) | 2.69 | 0.24 | 18.4 | -0.99 |
| Kronockij | 21 | 101 (65-137) | -1.35 | 0.15 | 30.9 | -1.25 |
| Malaja Sos'va | 23 | 105 (76-129) | -1.44 | 0.23 | 60.7 | -1.54 |
| Nurgush | 12 | 116 (81-146) | 6.37 | 0.26 | 9.8 | 0.15 |
| Olekminskij | 19 | 113 (105-119) | 0.04 | 0.36 | 24.3 | -1.84 |
| Pinezhskij | 36 | 97 (83-109) | -1.18 | 0.20 | 30.4 | -1.54 |
| Stolby | 53 | 101 (81-120) | 0.76 | 0.27 | 2.1 | -0.40 |
| Visherskij | 16 | 112 (100-138) | 3.11 | 0.25 | 13.7 | -1.61 |
| Visimskij | 30 | 103 (75-131) | 3.77 | 0.28 | 9.0 | -1.13 |
